# Post-treatment serum lactic dehydrogenase as a predictive indicator for distant metastasis and survival of patients with nasopharyngeal carcinoma

**DOI:** 10.18632/oncotarget.8480

**Published:** 2016-03-30

**Authors:** Jin Wang, Li Li, Bai-qiang Dong, Yu-jin Xu, Yuan-da Zheng, Zhong-wen Sun, Yang Yang, Yuan-Yuan Chen, Xiao-zhong Chen, Ming Chen

**Affiliations:** ^1^ Department of Radiation Oncology, Zhejiang Key Lab of Radiation Oncology, Zhejiang Cancer Hospital, Hangzhou, People's Republic of China; ^2^ Department of Ultrasonography, Zhejiang Cancer Hospital, Hangzhou, People's Republic of China; ^3^ Department of Oncology, Jining First People's Hospital, Jining, People's Republic of China

**Keywords:** serum lactic dehydrogenase, intensity-modulated radiation therapy, nasopharyngeal carcinoma, prognostic factor, metastasis

## Abstract

**Purpose:**

To examine the function of serum lactic dehydrogenase (SLDH) level after intensity-modulated radiotherapy (IMRT) as a predictive factor for and loco-regional relapse free survival (LRFS), distant metastasis-free survival (DMFS), disease free survival (DFS), and overall survival(OS) among patients with *in-situ* nasopharyngeal carcinoma (NPC).

**Results:**

Compared with the normal pt-SLDH group, elevated pt-SLDH demonstrated significant lower DMFS (46 versus 66 months, hazard ratio (HR) 4.07, 95% CI 2.43–6.80, *p* < 0.001), DFS (46 versus 63 months, HR 2.78, 95% CI 1.70–4.53, *p* < 0.001), and OS (54 versus 66 months, HR 2.93, 95% CI 1.65–5.23, *p* < 0.001). Distant metastasis were observed in 32.8% (20/61) patients with elevated pt-SLDH, and 8% (54/678) in normal SLDH (odds ratio (OR) 6.13, 95% CI 3.35–11.18, *p* < 0.001). COX regression showed that pt-SLDH was an independent prognostic factors for OS (HR 2.91, 95% CI 1.57–5.41, *p* < 0.001), DMFS (HR 4.21, 95% CI 2.51–7.07, *p* < 0.001), LRFS (HR 2.53, 95% CI 1.22–5.24, *p* < 0.001), and DFS (HR 2.81, 95% CI 1.72–4.59, *p* < 0.001).

**Materials and Methods:**

The records of 739 *in-situ* NPC patients admitted to Zhejiang Cancer Hospital between January 2007 and May 2012 were retrospectively reviewed. The relationships between post-treatment SLDH (pt-SLDH) and LRFS, DMFS, DFS, and OS were analyzed.

**Conclusions:**

Our finding indicated that elevated pt-SLDH could be a simple available prognostic indicator for distant metastasis and survival for *in-situ* NPC patients.

## INTRODUCTION

Nasopharyngeal carcinoma (NPC) has been considered as a rare type of cancer globally yet it is relatively frequent in some regions, including some locations of South-Eastern Asia and a number of provinces in South-Eastern China. Intensity-modulated radiotherapy (IMRT), which can deliver high radiation dose to the tumor but render little radiation to surrounding tissues, is becoming a standard radiotherapy (RT) technique for NPC [1, 2]. It is increasingly reported that IMRT alone or in combination with chemotherapy could improve loco-regional control and overall survival. However, 19% to 29% of patients have been found to have distant metastasis with controlled loco-regional lesion [3, 4]. To date, distant metastasis is still the major cause of mortality among NPC patients [5]. It is desirable to identify patients with higher risk at distant metastasis and unfavorable prognosis.

Lactate dehydrogenase (LDH), which reversibly transforms pyruvate to lactate under hypoxic conditions, is a tumor product in cancer patients. Serum LDH (SLDH) has also shown to be predictive of treatment outcome in various types of tumors [6, 7]. This is probably associated with hypoxia-mediated radio-resistance and the upregulation of metastasis-associated genes [6–8]. Multiple studies reported that SLDH prior to treatment was a predictive indicator for survival or distant metastasis among patients with NPC [8–17]. However, very limited research had been performed to explore the clinical significance of SLDH during follow-up period.

The objective of this study was to examine the relationship between SLDH measured during the follow up period (pt-SLDH) and locoregional relapse free survival (LRFS), distant metastasis-free survival (DMFS), and overall survival (OS) in patients with *in situ* NPC after IMRT.

## RESULTS

### Patient characteristics

The characteristics of the patients including age, gender, pathologic type according to the World Health Organization (WHO) classification, and AJCC stage distribution were outlined in Table 1. Totally 739 patient records were included in the study. The median age of the patients was 49 years old ranging from 18 to 81. The median overall RT treatment time was 44 days, and 84.8% of patients completed RT within 7 weeks. Six hundred and eighty-six patients received combined cisplatin-based concurrent chemotherapy. Six hundred and seventy-two patients received induction chemotherapy. One hundred and eighty-seven patients received adjuvant chemotherapy.

**Table 1:** Patient characteristics (*n* = 739 patients)

Characteristics	No of patients (%)*
Age, years	
≤ 50	415 (55.1)
> 50	324 (43.8)
Gender	
Male	515 (69.7)
Female	224 (30.3)
Pathologic type (WHO 1992)	
I	65 (8.8)
II and III	674 (91.2)
T category (AJCC 2009)	
T1	68 (9.2)
T2	141 (19.1)
T3	331 (44.8)
T4	199 (26.9)
N category (AJCC 2009)	
N0	79 (10.7)
N1	221 (30.7)
N2	351 (47.5)
N3(N3a and N3b)	82 (11.1)
Stage (AJCC 2009)	
I	9 (1.2)
II	78 (10.6)
III	396 (53.6)
IV a	174 (23.5)
IV b	82 (11.1)

Abbreviations: WHO, World Health Organization; AJCC, American Joint Committee on Cancer.

* Value may not add to 100% because of rounding.

### Overall survival

**Figure 1:**
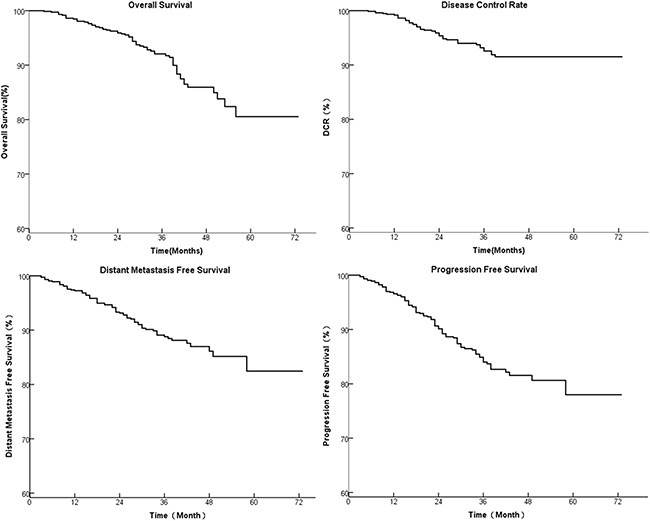
The 1, 3, 5 year survival of 739 patients with stage I to IVb NPC treated by IMRT (**A**): OS, (**B**): LRFS, (**C**): DMFS, and (**D**): DFS.

The follow-up time ranged from 3 to 72 months (median 34 months). The cumulative survive rate of 1, 3, 5 year was 97%, 92%, 81%, respectively (Figure 1A). The LRFS rate at 1, 3, 5 year was 99%, 93%, 92%, respectively (Figure 1B). The DMFS rate at 1, 3, 5 year was 97%, 88%, 82%, respectively (Figure 1C). The DFS rate at 1, 3, 5 year was 96%, 84%, 78%, respectively (Figure 1D).

### Correlation of pt-SLDH with prognosis

**Figure 2:**
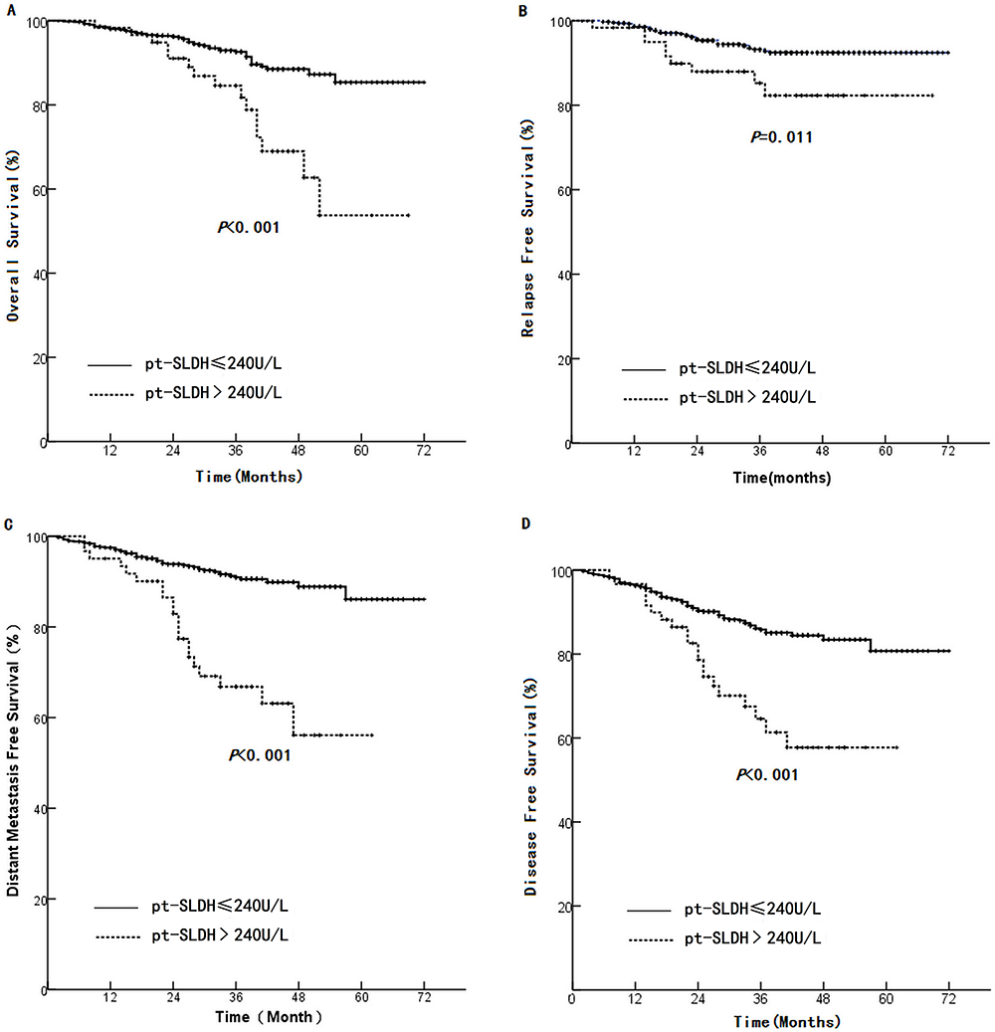
Comparison between high and normal pt-SLDH in (A): OS, (B): LRFS, (C): DMFS, and (D): DFS

The average peak pt-SLDH level was 204.9±161.3U/L ranging from 91-3278U/L. Patients with elevated pt-SLDH (> 240 U/L) demonstrated a significant lower OS compared to those with normal pt-SLDH (< = 240 U/L) (54 months versus 66 months, HR 2.93, 95% CI 1.65–5.23, *p* < 0.001, Figure 2A). Also, significant shorter LRFS (60 months versus 68 months, HR 2.49, 95% CI 1.21–5.16, *p* = 0.011, Figure 2C), DMFS (46 months versus 66 months, HR 4.07, 95% CI 2.43–6.80, *p* < 0.001, Figure 2C), and DFS (46 months versus 63 months, HR 2.78, 95% CI 1.70–4.53, *p* < 0.001, Figure 2D) were found in elevated pt-SLDH group compared to the normal group.

**Figure 3:**
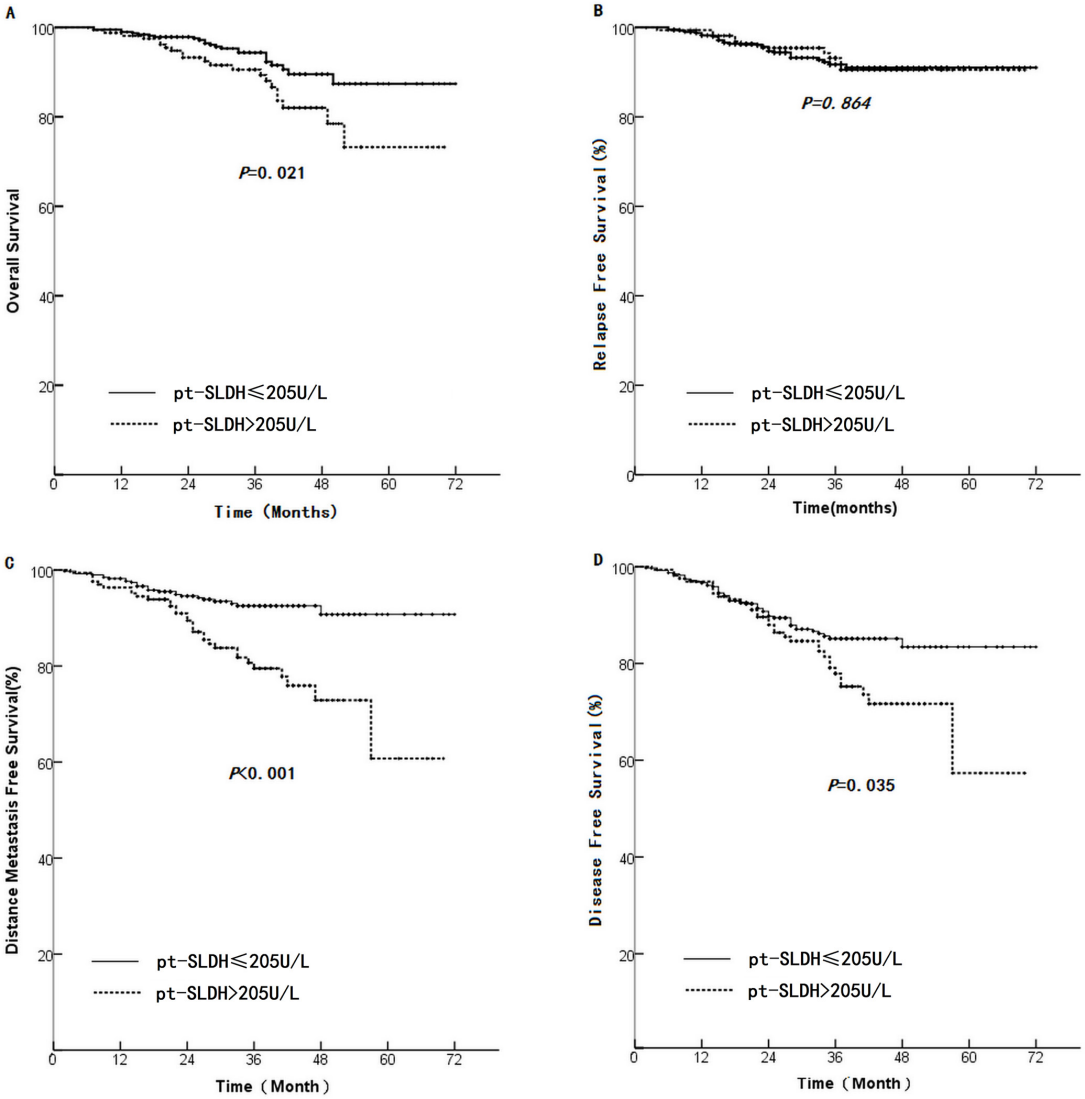
Comparison of survival rate between patients with pt-SLDH > 205 U/L and those with pt-SLDH < 205 U/L (**A**): OS, (**B**): LRFS, (**C**): DMFS, and (**D**): DFS.

We performed another analysis by dividing the patients into two groups based on the median pt-SLDH level of 205.0 U/L. We found that the group of ≤ 205.0 U/L had significant prolonged median OS (67 months versus 61 months, HR 2.00, 95% CI 1.09–3.53, *p* = 0.021, Figure 3A), median DMFS (68 months versus 57 months, HR 2.84, 95% CI 1.69 – 4.78 *p* < 0.001 Figure 3C), and median DFS (64 months versus 56 months, HR 1.60, 95% CI 1.03–2.48 *p* = 0.035 Figure 3D) compared with the group of > 205.0 U/L. However, no significant difference in RFS was found between the two groups (median: 68 months versus 66 months, *p* = 0.864, Figure. 3B).

### Correlation between the SLDH change before and after the treatment with prognosis

**Figure 4:**
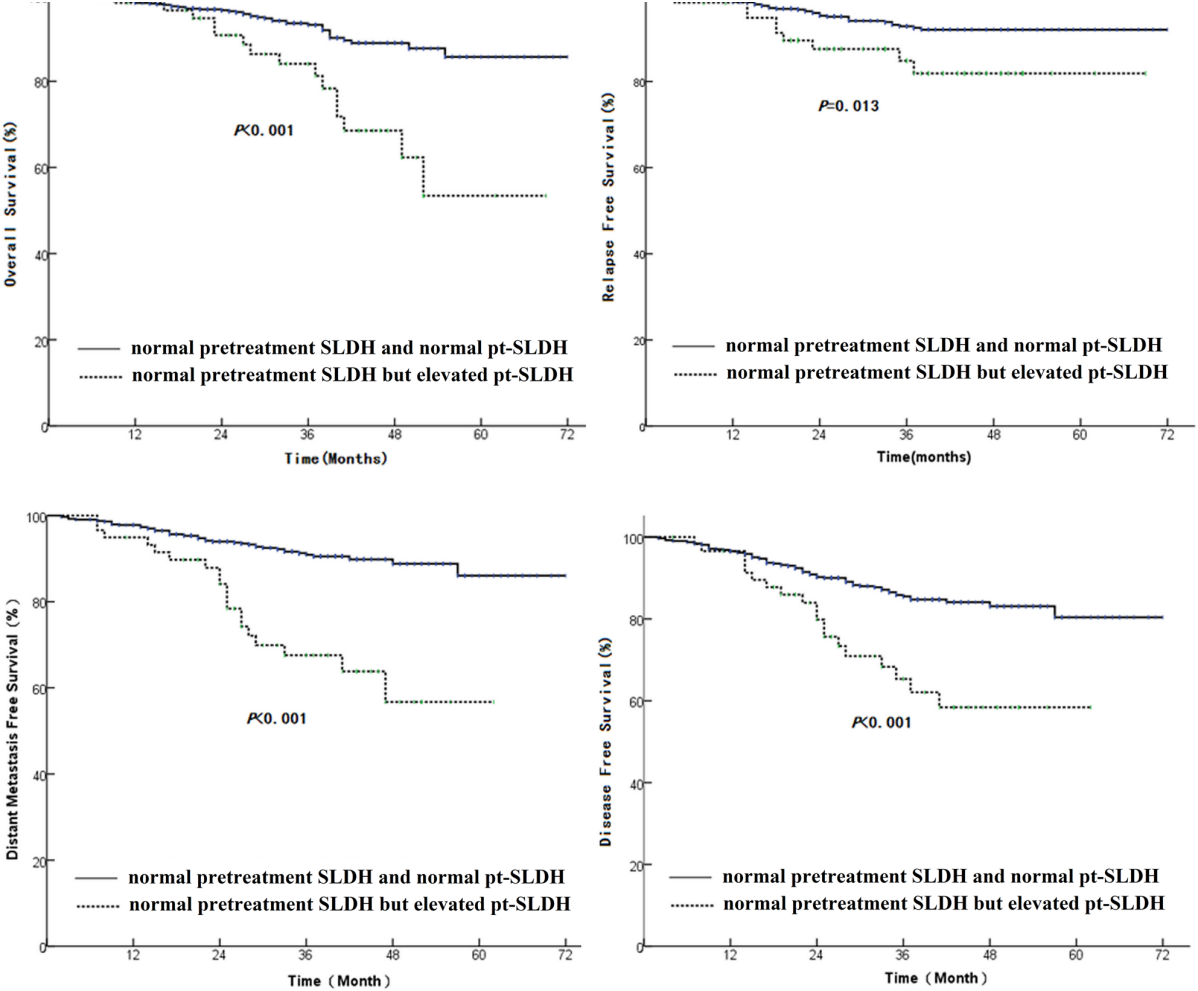
Comparison of survival rate between patients having normal pretreatment SLDH and normal pt-SLDH with those having normal pretreatment SLDH but elevated pt-SLDH (**A**): OS, (**B**): LRFS, (**C**): DMFS, and (**D**): DFS.

The average SLDH level before treatment was 175.7 ± 43.11 U/L (ranging from 9–528 U/L), which was independent of patient's age, gender, T stage, N stage, and AJCC stage status. Univariate analysis found that pretreatment SLDH has no significant correlation with survival rate. However, we found that patients with normal pre-treatment SLDH and elevated pt-SLDH had poor OS, LRFS, DMFS, DFS (Figure 4).

**Figure 5:**
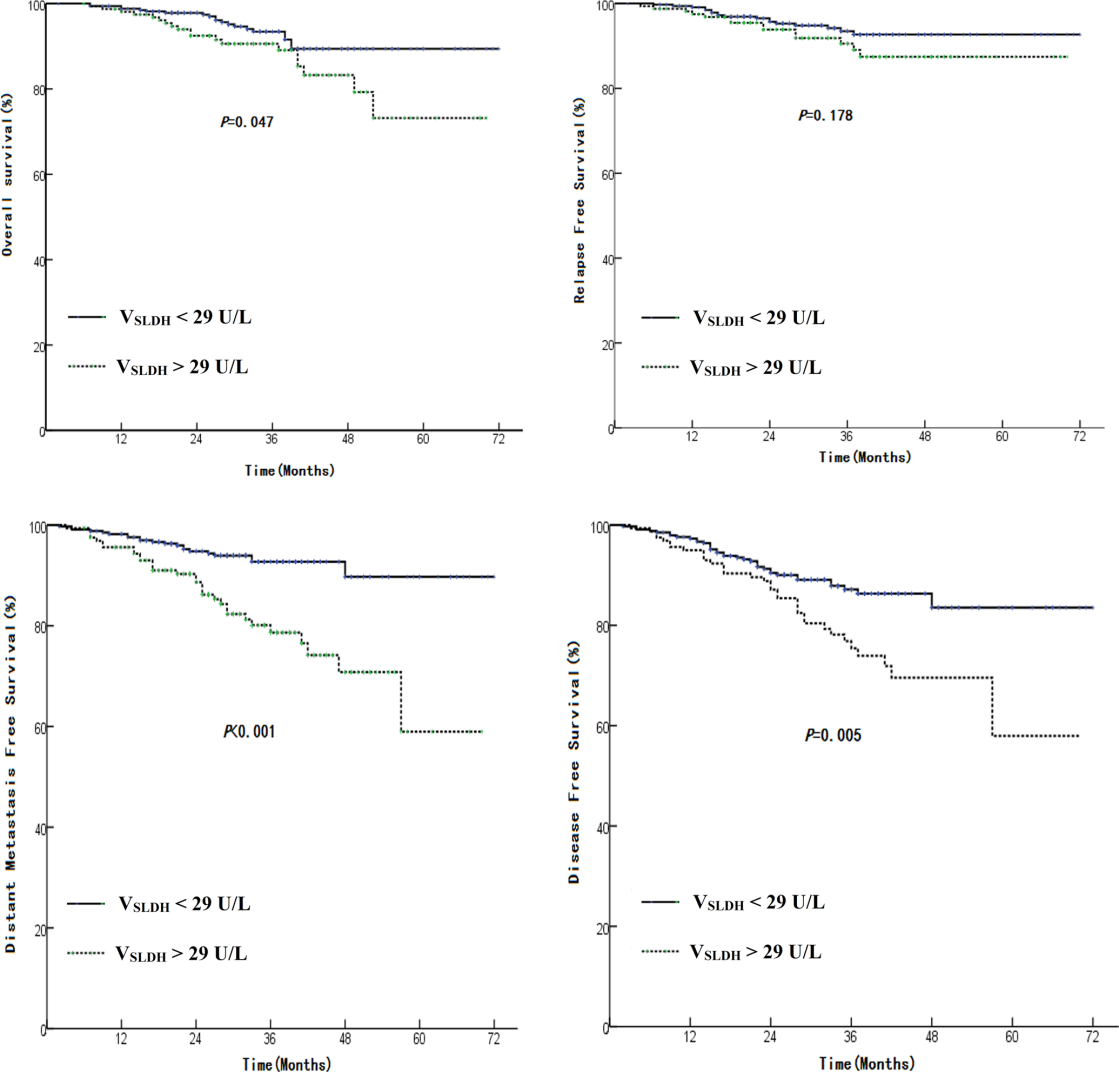
Comparison of survival rate between patients having a V_SLDH_ < 29 U/L with those have a V_SLDH_ > 29 U/L V_SLDH_ = pt-SLDH – pretreatment SLDH. (**A**): OS, (**B**): LRFS, (**C**): DMFS, and (**D**): DFS.

We further analyzed the effect of the variance of SLDH between pre- and post- treatment (V_SLDH_). The V_LDH_ from the 739 patients ranged from −381 to 3071 (Mean 28.51 ± 168.95). Then we rounded the mean value and used it to separate the patients into two groups: V_SLDH_ > 29 U/L and ≤ 29 U/L. The group with V_SLDH_ > 29 U/L had significant lower median OS (61 months vs 67 months, HR 1.87, 95% CI 1.00–3.51, *p* = 0.047), DMFS (56 months vs 67 months, HR 3.09, 95% CI 1.78–5.38, *p* < 0.001), and DFS (55 months vs 64 months, HR 1.93, 95% CI 1.21–3.08, *p* = 0.005) than those with VSLDH ≤ 29 U/L, but there is no significant deference in LRFS between two groups (*p* = 0.178, Figure 5).

### Correlation of pt-SLDH with metastasis

Distant metastases were found in 20 of the 61 (32.8%) patients with elevated pt-SLDH, and in 54 of the 678 (8%) patients with normal pt-SLDH (odds ratio (OR): 6.13, 95% CI: 3.35–11.18, *p* < 0.001).

Elevated pt-SLDH were found in 20 of 74 (27.0%) patients who developed metastasis during follow-up, among whom 20% patients were found to have elevated pt-SLDH before metastasis were diagnosed.

### Multivariate analysis

COX regression showed that age, overall RT time, pt-SLDH, and tumor stage were independent influence factors for overall survival (Table 2). In multivariate analysis of DMFS, LRFS, DFS, elevated pt-SLDH was associated with a high risk of distant metastasis (HR 4.21 95% CI 2.51–7.07 *p* < 0.001), relapse (HR 2.53 95% CI 1.22–5.24 *p* < 0.001), and disease progression (HR 2.81 95% CI 1.72–4.59 *p* < 0.001).

**Table 2:** COX regression of clinical factor on overall survival (739 patients)

Parameters	B	S.E	Wald	*P*	Exp (B)	95% CI
Stage	0.436	0.217	4.056	**0.044**	1.547	1.012–2.365
pt-SLDH ≥ 240U/L	1.123	0.313	12.849	**< 0.001**	3.074	1.684–5.680
RT time	0.058	0.020	8.323	**0.004**	1.06	1.019–1.103
Age	0.050	0.013	13.839	**< 0.001**	1.051	1.024–1.079

## DISCUSSION

Our previous studies showed that inhibition of LDH induced G2/M cycle arrest, apoptosis, and autophagy in NPC and NSCLC cells [18, 19]. In this study, we investigated the prognostic value of pt-SLDH for NPC patients through retrospective analysis of clinical data. We found that pt-SLDH was correlated significantly with risk of distant metastasis, survival time, and local relapse, especially when pt-SLDH was 29 U/L or higher than pre-treatment level. Also, significant lower DMFS, DFS, OS were observed in patients with elevated pt-SLDH than those of normal pt-SLDH, disregard of the pretreatment SLDH level. Furthermore, elevated pt-SLDH was associated with high rate of distant metastasis, and could be a predictor for metastasis before it was identified. Our study indicated that pt-SLDH might be a good marker for monitoring the disease progress and treatment efficacy.

Our result was echoed by some other studies that indicated pt-SLDH being linked with failure of radiotherapy and chemotherapy in sarcomas, lymphomas, and carcinomas [8]. Jin *et al.* also observed poor survival in metastatic NPC patients with normal pre-treatment and elevated pt-SLDH [5]. The SLDH level decreased while patients were in remission, and increased while patients were in relapse.

Many studies showed that SLDH had been identified as a prognostic indicator for NPC patients [9–15]. In locally advanced NPC patients, elevated pretreatment SLDH has been reported as a risk factor for distant metastasis, or local relapse [8, 10, 16]. Wan *et al*. analyzed a phase III randomized controlled study and found that elevated pretreatment SLDH correlated with poor OS, DFS, LRFS, and DMFS in patients with locally advanced NPC [17]. Zhang *et al*. found that pre-treatment SLDH could be prognostic for OS and DMFS in NPC patients with a positive family history [20].

Unfortunately, there is only a few publications about the pt-SLDH. Our study is the only study with largest case number on prognostic value of pt-SLDH for *in-situ* NPC. The mechanisms of the observation in this study was not clear. Elevated SLDH might not only be a reflection of heavier tumor burden, but also promote tumor progression by influencing the tumor metabolism and microenvironment. LDH is an important enzyme in energy production under hypoxic conditions and plays critical roles in regulating carcinogenesis, tumor metabolic reprogramming, and tumor angiogenesis. The association between LDH and oncogenic anaerobic glycolysis, or the Warburg effect, could facilitate tumor growth and metastasis. This metabolic reprogramming is regulated by HIF-1α, through the transcriptional activation of key genes encoding metabolic enzymes including LDH. This process is closely associated with an increased risk of invasion, metastasis, and mortality [20, 21]. It is reasonable to speculate that high SLDH could result in poor survival through aggressive tumor progression.

Many studies have reported that age was an independent factor for prognosis of NPC, and we observed the same result [22, 23]. The mechanism is still not clear. No correlation was found between age and pt-SLDH or the V_SLDH_. Delayed diagnosis of NPC could be one of the causes, based on the observation that older patient had higher stage of malignancy.

There are certain limitations in this study. As a retrospective study, certain bias may existed in patient selection and application of RT and chemotherapy. LDH has five isoenzymes, and each plays different role in tumor growth and distant metastases. In this study, our data could not distinguish the serum level of any particular isoenzyme, which could be even more important for further investigation.

In conclusion, our finding indicated that elevated serum LDH after radiotherapy provided could be a simple available prognostic marker for distant metastasis, relapse and survival of *in-situ* NPC patients. In clinical practice, physician should pay more attention to those with abnormal pt-SLDH, or whose V_SLDH_ is above 29U/L. More examination should be ordered for metastasis detection. Future study could focus on the identification of the isoenzyme of LDH and/or in combination with other tumor marker, such as EBV, for more sensitive and specific prediction [24, 25].

## MATERIALS AND METHODS

### Patient

The protocol was approved by the Institutional Review Board (IRB) of Zhejiang Cancer hospital. The NPC patient records hospitalized in Zhejiang Cancer Hospital between January 2007 and May 2012 were reviewed. Data from patients who met the following criteria were selected for further analysis: 18 or older; histologically diagnosed as stage I–IVb NPC (based on the American Joint of Cancer Committee 2009 stage system); complete pre- and post-treatment SLDH record; more than 3 month follow-up; Karnofsky Performance Scores > 70; normal renal and cardiac function; no complications as active hepatitis, tuberculosis, acute infections, pneumonia, and pulmonary infarction during treatment and follow-up period; and has been treated with a full course of IMRT.

### Radiotherapy

All patients received curative IMRT as described by Chen *et al.* [26, 27]. Briefly, patients were positioned in supine and immobilized from head to neck with a thermoplastic mask in both CT simulation and treatment delivery. The planning CT was acquired with a slice thickness of 3 mm and was transferred to a treatment-planning system (PINNCAL 8.0/9.2, PHILIPS Corp.) for IMRT planning. The gross tumor volume (GTV) was defined according to diagnostic MRI, CT and physical examination. The clinical target volume 1 (CTV1) was defined as the nasopharynx GTV plus a 5 to 10 mm margin, while CTV2 was the CTV1 plus a 5 to 10 mm margin except 2–3 mm posteriorly, including the elective neck area. Planning target volumes (PTV) for all GTVs and CTVs were generated automatically by adding a non-uniform margin to account for the immobilization and localization uncertainties (≤ 3 mm). Organs at risk (OAR) were delineated from the planning CT. IMRT plans were generated with a prescribed dose of 69 Gy in 30 fractions to the GTV, 60–64 Gy to CTV1 and 50–54 Gy to CTV2. The doses to OARs were minimized without sacrificing target coverage.

### Chemotherapy

Patients received induction chemotherapy, concurrent or adjuvant chemotherapy as described by Chen et al. [26, 27]. Briefly, the induction chemotherapy consisted of two or three cycles of docetaxel 75 mg/m^2^ and cisplatin 75 mg/m^2^ every three weeks, or cisplatin 80 mg/m^2^ on Day 1 and 5-fluorouracil 4.0 g/m^2^ by continuous intravenous infusion on 120 hours every three weeks. For concurrent chemotherapy, cisplatin was given with 80 mg/m^2^ every three weeks or 40 mg/m^2^/week during the course of RT. Adjuvant chemotherapy plan included cisplatin 80 mg/m^2^ on day 1 and 5-fluorouracil 4.0 g/m^2^ by continuous intravenous infusion on 120 hours every three weeks for 3 to 4 cycles [26, 27].

### Follow-ups

Nasopharynx was observed under indirect laryngoscopy or fiberoptic pharyngorhinoscopy for disease remission every week during RT, and 4 weeks and 12 weeks after RT. After the completion of entire treatment, patients were followed every two months in the first 6 months, every three month in the following 7 to 40 months, and once a year afterwards. The examined items in each follow up included blood routine and biochemical examination (including SLDH), chest X-ray, abdominal B type ultrasonic examination, head and neck CT or MRI, and other examinations when necessary [26, 27].

### SLDH measurement

SLDH was measured prior to the radio- and chemo-therapy and at each visit time during follow up period by Hitachi Modular 7600 Chemistry Analyzer. The optimized standard method recommended by the German Society of Clinical Chemistry was employed. A value of > 240 U/L was considered abnormal [28].

### Statistical analysis

The chi-square test or Fisher's exact test (when expected count < 5) was used for comparison of categorical data. K-M regression was used to determine independent factor for survival. Receiver operating characteristic curves (ROCs) were used to calculate sensitivity and specificity at each cut off value of the parameters. The optimal cut off value is achieved when the sum of the sensitivity and specificity reaches a maximum value. COX regression multivariate was performed for each of the clinical end points (OS, DMFS, LRFS, DFS) in order to evaluate the potential significant prognostic factors. All statistical tests were two-sided and a *p* value of less than 0.05 was considered statistically significant. The data processing and statistical analyses were performed using SPSS software (Version 15.0, SPSS Inc, Chicago, IL).
